# Lifestyle and Dietary Behaviors among Saudi Preschool Children Attending Primary Health Care Centers, Eastern Saudi Arabia

**DOI:** 10.1155/2014/432732

**Published:** 2014-07-10

**Authors:** Magdy A. Darwish, Ghadeer Al-Saif, Suha Albahrani, Amr A. Sabra

**Affiliations:** ^1^Department of Family and Community Medicine, University of Dammam, Saudi Arabia; ^2^Qatif Primary Health Care, Ministry of Health, Eastern Province, Saudi Arabia; ^3^Dammam Primary Health Care, Ministry of Health, Eastern Province, Saudi Arabia

## Abstract

*Objective*. To study life styles and dietary behaviors among Saudi preschool children (1–5 years) attending primary health care centers (PHCCs) in Dammam and Qatif areas, eastern province, Saudi Arabia. *Material and Methods*. Cross-sectional study. Data were collected using structured, interviewer-filled questionnaire. Children and their mothers were encountered during their well-baby clinic visits. A total number of 300 preschool children and their mothers were interviewed during study period. *Results*. Unsatisfactory areas include smoking fathers (32%), smoking in front of children (11.3%), overweight and obesity among mothers (60.3%), noncompliance using seat belts for both parents (56.3%) and children (68%), children watching television (T.V) more than 2 hours (50%), adherence to exclusive breast feeding (only 20.7%), and late solid food introduction (65.3%). Frequent intake of unhealthy food items was 26%, 25%, and 24% for pizza, burger, and soft drinks. Unfortunately frequent intake of the following unhealthy food items was high: biscuits, deserts/chocolates, and chips which was 78%, 67%, and 72%, respectively. *Conclusion*. This study provides benchmark about the current situation. It provides health care workers and decision makers with important information that may help to improve health services.

## 1. Introduction

Recent research has begun to focus on effects of family and social influences on children's lifestyle and eating patterns [1]. Key components of pediatric lifestyle include starting with exclusive breast feeding, optimal nutrition, maintaining appropriate weight, moderate physical activity, optimum sleep duration, and avoidance of long hours of watching television (TV) [2–4]. Cardiovascular risk factors start in early childhood with fatty streaks evident in the arteries of children [4]. Recently, levels of blood cholesterol and triglycerides have been found to be increasing in children while the high density lipoproteins decreased [4]. Moreover Research has demonstrated that both the physical and social environment strongly affect the eating patterns of children [1]. Social environment, including various socioeconomic and sociocultural factors, influence the types of foods that children eat [1]. Parents actually have potential and powerful role in behavioral change strategies which aim to improve the lifestyle behaviors of young children [2]. Parental obesity, low parental educational level, low total family income, long hours of TV watching, absence of breastfeeding, and physical inactivity were significantly associated with childhood overweight/obesity [1]. Low maternal educational level and allowing children to watch TV more than 2 hours were also associated with unhealthy snaky pattern [3]. Overeating-type eating style and sedentary activities are observed frequently in the children from obese/overweight families [5]. This is associated with the fact that these children had a higher preference for fatty foods with a lower liking for vegetables [5]. The mother's work status played a significant role in the early termination of breastfeeding [6]. Early return to work and lack of maternity leave contribute to early cessation of breastfeeding or lack of exclusive breastfeeding [4]. Long hours of maternal employment, rather than lack of money, may impede children's access to healthy foods and physical activity but there was no evidence for that association with paternal work [7]. Family income often affects accessibility to healthy food that is why the lower socioeconomic status acts as a barrier to the fruit and vegetables intake and makes the intake of fat higher compared to children in relatively higher socioeconomic groups [1]. However, to fulfill this role, parents need to have the necessary knowledge and motivation to assimilate dietary guidelines [2]. Some day care centers play an important role in the development of children's eating habits by focusing on issues such as providing and making healthful food choices like fruit and vegetables [2]. Playing is very important for child development because it contributes to the cognitive, physical, social, and emotional well-being and should be included along with academic and social-enrichment opportunities [8]. In addition, sleep duration is important for children. An inverse association was observed between sleep duration and the risk of developing childhood overweight/obesity [2]. Promoting healthier eating patterns among children requires a multifaceted approach targeting children, parents, families, and schools. Interventions aimed at improving children's nutrition need first to address variety of social and physical factors that could influence children's eating patterns. Clearly, this area is rich and needs researches to address multiple influences on children's eating patterns and life style.

## 2. Methodology

This cross-sectional study was conducted in PHCCs in Qatif and Dammam areas, eastern region, Saudi Arabia. Study population included Saudi preschool children (1–5 year old) attending chosen PHCCs and their mothers/caregivers. Systemic random sampling technique was used by choosing every second PHCC from lists of PHCCs provided by ministry of health in both Dammam and Qatif areas. A total of 13 centers out of the 27 centers in Qatif and 11 centers out of the 22 centers in Dammam were chosen where children and their caregivers were encountered during their well-baby clinic visits. All preschool children and their mothers attending the chosen centers were interviewed on days of study visit. A total number of 326 preschool children and their mothers were interviewed during study period.

Data were collected using structured, interviewer-filled questionnaire which has been designed by the researchers after reviewing the recent literature and similar questionnaires and based on the objectives of the study putting in consideration sociocultural backgrounds. The questionnaire was divided into four parts: First part included sociodemographic data of the care givers like age, sex, marital status, occupation, and so forth. Second part included questions to assess practice of healthy styles among parents for example smoking, use of seat belt … and so forth. Third part included child socio-demographic data and child lifestyles, for example, age, sex weight, height, rank, siblings, attending nursery, TV watching, physical activity, sleeping pattern, breast feeding and weaning, and so forth. Fourth part included questions about dietary behaviors of children, for example, number of meals, number of snacks, and so forth. Consumption of different types of commonly available healthy and unhealthy food items (19 item) was evaluated using simplified Arabic frequency questionnaire which has high internal consistency (Cronbach's alpha scores of >0.74).

A pilot study was conducted on 20 patients—different from the target group—to check understanding and applicability of the questionnaire. Based on the results, some linguistic modifications of questions were made to avoid confusion about questions and make easier understanding and interpretation by participants. Questionnaire was then reviewed by researchers, two of them have Saudi Arabian slang: one from Qatif and the other from Dammam, before and after pilot study for necessary linguistic modifications of some confusing words. Questionnaires were reviewed for completeness and invalid questionnaires were excluded giving a total of 300 valid questionnaires.

Questionnaire was validated after modification. Questionnaire was reviewed by 2 faculties, revised questionnaires were compared and necessary modifications were made before finally approved by the reviewers.

The participants were approached in well baby clinics of chosen primary health care centers. They were met and explained purpose of the study, reassured that questionnaires are anonymous, and informed that collected data will kept confidential and used only for study purpose. The questionnaire was explained to them and all their questions were answered before obtaining their informed consent.

The data were coded, entered, and analyzed in a personal computer using statistical package for social sciences (SPSS) software version 16. Data were presented using descriptive statistics in form of frequencies and percentages for qualitative variables and mean and standard deviation (SD) for quantitative variables. Chi-square test and/or logistic regression analysis were used as appropriate to determine association.

The study was approved by ethical committee of Postgraduate Saudi Board Program, Eastern Province. All necessary approvals from ministry of health including ethical approval were obtained.

## 3. Results 

A total of 300 valid questionnaires were obtained representing 300 preschool children and their mother's responses. The mean age for study population mothers in years was 31.2 ± 5.6 SD. All care givers in this study were children's mothers. Nearly half of the mothers (47.7%) were in age range 30 to 40 years, Most of the sample was married (98.3%) and 71.3% of them have very good relationships with the fathers. Educational level of the mothers and fathers in this study was university graduation or above in 50% and 48.3%, respectively, and more than half of the mothers (52.7%) were housewives (Table 1).

Some life style characters of parents of study group including smoking habits, use of seat belts, and body mass index of study mothers are illustrated in Table 2 while Table 3 shows the Sociodemographic characteristics and health behaviors of the children participating in the study including age, sex, birth order, number of siblings, and child morbidity.  Table 4 illustrates health behaviors of the children including playing patterns, TV watching, modes of breast feeding and weaning, nursery/kindergarten attendance, and meal and snacking patterns. Distribution of studied children according to their nutritional status showed that all anthropometric measurements lie within normal range for study population children. Weight and height were measured and then weight for age, height for age, and weight for height were calculated for all children participating in this study using standard age suitable charts. All charts used were latest Saudi growth charts used by MOH (Table 5). Factors related to consumption of healthy food items among study population preschool children are shown in Table 6. Consuming more vegetables was significantly related to number of meals per day (*P* = 0.00), while frequency of fruit consumption was more in children whom mothers were housewives (*P* = 0.00), children living in nuclear family (*P* = 0.03), and children eating more snacks (*P* = 0.00). Legumes consumption was more among those living in Dammam (*P* = 0.00) and in children consuming more meals (*P* = 00.04). 60.20% of children who consume frequent amount of legumes were taking three meals/day. Factors significantly affecting consuming unhealthy food items in preschool study population children are shown in Tables 7 and 8. Children rising in nuclear families were consuming pizza and burger more frequently than children rising in extended families (75.90% and 81.60%, resp.), while children of families living in Qatif were consuming burger and soft drinks less frequently. Soft drinks consumption was also less frequent in children who eat more meals (*P* = 0.002) and among children not attending and those attending less months in nursery or kindergarten (*P* = 0.015) (Table 7). Children in families with higher total monthly income were consuming more amounts of biscuit (*P* = 0.049). Also children who were not attending nursery or kindergarten where consuming more amount deserts/chocolate (*P* = 0.04). Watching T.V more than 2 hours was associated with more frequent consumption of deserts/chocolate and chips (*P* = 0.025) and (*P* = 0.006), respectively. In addition, chips consumption was more frequent in children of housewife mothers (*P* = 0.037) (Table 8).

## 4. Discussion 

Frequency of smoking among fathers was 32%, while 1.3% of mothers were smokers. 11.3% of children were passive smokers due to father or mother smoking. These percentages are in the ranges of the national data. Bassiony (2009) in his study showed prevalence of smoking in Saudi males ranges from 13 to 38% (median = 26.5%), while in Saudi females it ranges from 1 to 16% (median = 9%) [9]. A study done in Germany shows that 28.5% of fathers and 20.7% of mothers were smokers [10]. Characteristically in our study prevalence of smoking among mothers was low in contrast to smoking among fathers.

Regarding fastening seatbelt, 43.7% of parents who participated in our study used to fasten seatbelt. A study done in eastern province in Saudi Arabia shows that the usage of seatbelts ranged from 17 to 100% with a mean of 56.8% [11]. Another study done in Riyadh showed that 62.4% of participants were fastening seatbelt [12]. A regional study done in gulf area shows that the median percentage of the seatbelt noncompliance was significantly higher in the Gulf countries (52%) compared with the high-income countries (14.5%) (*P* < 0.001) [13]. Fastening seat belt among parents was less in our study than national, regional, and international studies. Only 34% of parents of children in our study were fastening seatbelt compared to only 24% in Jeddah [14], while a study done in USA shows that 91% of children were strained in the car [15].

This means that fastening seatbelt awareness and rules in our country is underdeveloped and needs improvement.

About weight in our study, mothers showed that 36.7% have normal body weight, 32% were overweight, and 28.1% were obese which is similar to results of the study of Al-Nozha done in KSA 2005 that showed overall prevalence of obesity 35.6% (95% CI: 34.9–36.3), while severe (gross) obesity was 3.2% [16]. In Bahrain, approximately 32% of women were obese (BMI ≥ 30) [17]. Generally speaking there is a significant increase in the incidence of obesity with a prevalence of 2%–55% in adult females and 1%–30% in adult males over the Arabic-speaking countries [18].

In our study population, 50% of the children watch T.V for less than 2 hours and the remaining 50% watch for 2 hours or more. The American Academy of Pediatrics (AAP) recommends that children 2 years and older watch <2 hours of television per day and that children younger than 2 years watch no television [19]. However, 26% of US children watched 4 or more hours of television per day and 67% watched at least 2 hours per day [20]. In our study TV watching was associated with more frequent consumption of unhealthy items (chips and chocolate). Studies have shown unhealthy pattern of diets with TV watching with more unhealthy items and less healthy items intake and less frequent meals [21–23].

Time spent in playing by most children in our study was satisfactory. Only 11% of children spend less than 2 hours in playing while 42.7% spend 2 to 4 hours in playing and 46.3% spend 5 hours or more in playing. National Association for Sport and Physical Education (NASPE) guidelines recommend for preschool children to (engage in at least 60 minutes—up to several hours—of unstructured physical activity each day and should not be sedentary for more than 60 minutes at a time, except when sleeping) [24].

Although the world health organization (WHO) recommends exclusive breast feeding up to 6 months duration [25], we found in our study that only 20.7% of the mothers exclusively breastfed their babies, 68.6% were on mixed feeding, 10.7% of the mothers never breastfed their babies, and 26.3% have less than 6 months duration of breast feeding. Prevalence of exclusive breastfeeding was extremely low in our population while partial breastfeeding was the trend for feeding in the first 6 months of life; similar results were concluded from many studies done in Saudi Arabia which was accompanied with rapid decline in lactation duration [26, 27].

In this study we found that 65.3% of children were introduced solid food at age of 6 month or more while 34.7% of children were introduced before the age of 6 months. This means that still there is misconception about the proper age of introduction of solid food. WHO recommends introduction of solid food around the age of 6 month [28], and recent studies show that late introduction of food was associated with increased risk of allergic sensitization to food [29, 30] and inhalant allergens [29].

Regarding frequency of consumption of different food items, consumption of the following healthy items was noticeably rare among study participants: red meat (16%), white meat (9%), fish (25%), beans (49%), eggs (11.7%), and nuts (49.7%).

Unfortunately consumption of the following unhealthy items was frequent (that is consumed most of the days) among study participants: pizza (26.4%), burger (25.3%), chocolate/desert (67%), butter-mayonnaise (25%), soft drinks (24%), juices (87%), chips (71.7%), and biscuits (78.3%).

In our group study, there was a positive relation between family income and biscuits eating. In 2003 Xiea B showed that increased fast-food consumption was independently associated with higher household incomes [31].

Children living in nuclear families were found to consume more unhealthy items (pizza and burger) than children living in extended families. As extended families has members from old generation whom consuming homemade food more as shown in a study done in USA [32].

Children living in Qatif (rural area) were consuming less unhealthy items (burger and soda) than those living in Dammam (urban area). Colić-Barić et al. (2004) showed that Consumption of fast food and soft drinks was more prevalent in urban than rural area [33].

Strangely legumes (healthy food item) intake was lower among children of Qatif area. This can be explained by high prevalence of G6PD deficiency in this area.

Children attending nursery were consuming less deserts/chocolate and drinking more soft drinks in our study. Russell and Worsley (2007) showed that there were no significant associations between attending day care facility and children's food preferences [34].

In our study we found that increasing meal frequency was associated with less frequent soft drinks, more frequent vegetables and legumes and that children who consume more snacks consume more fruit.

Regular meals are associated with improved dietary intake among family members [35]. For example, several large studies have shown that regular family meals are strongly associated with increased consumption of fruits, vegetables, and grains and also linked with lesser consumption of fried or fatty foods and soft drinks or other unhealthy food choices [36–40].

Bellisle (2007) stated that having up to 3 snacks a day can have a positive, rather than a negative, impact on health [41]. The study of Pedersen et al. (2012) showed significant association between irregular breakfast, lunch and evening meal consumption and low frequency of fruit intake and vegetable intake [42]. Lazzeri et al. (2013) showed significant relation between low fruit and vegetable intake and irregular breakfast habits. Similarly, low fruit intake was associated with irregular snack consumption [43].

## 5. Conclusion 

Studying life styles, dietary behaviors, and factors that may affect them among preschool children provides benchmark about current situation. It provides health care workers and decision makers with important information that will help to improve health services especially by providing effective, preventive health education and health promotion services to improve situation in this vulnerable sector of the community.

## Figures and Tables

**Table 1:** Sociodemographic data of the parents of the children participating in the study.

Sociodemographic characteristic	Total (*n* = 300)	
	No	%
Age of the mother in years		
(i) <20	3	1.0
(ii) 20–<30	127	42.3
(iii) 30–<40	143	47.7
(iv) ≥40	27	9.0
Mean age in years ± SD = 31.2 ± 5.6		
Marital status of the mother		
(i) Married with father	295	98.4
(ii) Divorced	4	1.3
(iii) Widow	1	0.3
Educational level of the mother		
(i) Illiterate/Read and write	6	2
(ii) Primary school	13	4.3
(iii) Intermediate	15	5.0
(iv) High or diploma	116	38.7
(v) University or more	150	50.0
Educational level of the father		
(i) Illiterate/Read and write	4	1.3
(ii) Primary school	11	3.7
(iii) Intermediate	24	8.0
(iv) High or diploma	116	38.7
(v) University or more	145	48.3
Total family income in Saudi Riyals (SR)		
(i) <2000	10	3.3
(ii) 2000–4999	54	18.0
(iii) 5000–9999	104	34.7
(iv) 10000–14999	59	19.7
(v) 15000 or more	73	24.3
Relationship between parents (mother statement)		
(i) Very good	214	71.3
(ii) Good	5.9	19.7
(iii) Minor problems	20	6.7
(iv) Major problems	3	1.0
(v) Separated	4	1.3
Family type		
(i) Nuclear family	213	71.0
(ii) Extended family	87	29.0
Mother work		
(i) Housewife	158	52.7
(ii) Employed	142	47.3
father work		
(i) Not working	14	4.7
(ii) Manual worker	30	10.0
(iii) Professional	256	85.3

**Table 2:** Life style of parents of study group children.

Life style of the parents	Total (*n* = 300)	
	No	%
Smoking		
(i) Smoking father	96	32.0
(ii) Smoking mother	4	1.3
(iii) Smoking in front children	34	11.3
Fastening seat belt		
(i) Parents fastening seatbelt	131	43.7
(ii) Children fastening seatbelt	96	32.0
BMI of the mother		
(i) Underweight (<18.5)	9	3.0
(ii) Normal (18.5–<25)	110	36.7
(iii) Overweight (25–<30)	96	32.0
(iv) Obese class 1 (30–<35)	53	17.6
(v) Obese class 2 (35–<40)	24	8.0
(vi) Morbid obese (40 or more)	8	2.7

**Table 3:** Sociodemographic characteristics of the children participating in the study.

Sociodemographic characteristics of the children	Total (*n* = 300)	
	No	%
Child age in years		
(i) 1 year	43	14.3
(ii) 2 years	75	25.0
(iii) 3 years	52	17.3
(iv) 4 years	50	16.7
(v) 5 years	80	26.7
Child sex		
(i) Male	137	45.7
(ii) Female	163	54.3
Child order		
(i) 1st	111	37.0
(ii) 2nd	70	23.3
(iii) 3rd	35	11.7
(iv) 4th	36	12.0
(v) 5th or more	48	16.0
Number of siblings		
(i) No siblings	48	16.0
(ii) 1 sibling	75	25.0
(iii) 2 siblings	60	20.0
(iv) 3 siblings	43	14.3
(v) 4 siblings or more	74	24.7
Child attending nursery or kindergarten (KG)		
(i) Attending	86	28.7
(ii) Not-attending	214	71.3
Number of months attended in nursery or KG/year		
(i) 3–5 months	4	1.3
(ii) 6–8 months	45	15.0
(iii) 9–12 months	37	12.4
(iv) Not-attending	214	71.3
Number of hours attended in nursery or KG/day		
(i) 3–6 hours	64	21.4
(ii) 7–10 hours	22	7.3
(iii) Not-attending	214	71.3
Number of meals eaten in nursery or KG		
(i) 1 meal	57	19.0
(ii) 2 meals	27	9.0
(iii) 3 meals	2	0.7
(iv) Not-attending	214	71.3

**Table 4:** Health behaviors of the children participating in the study.

Health behaviors	Total (*n* = 300)	
	No	%
TV watching hours/day		
(i) <2 hours	150	50.0
(ii) ≥2 hours	150	50.0
Playing hours/day		
(i) <2 hours	33	11.0
(ii) 2–4 hours	128	42.7
(iii) ≥5 hours	139	46.3
Breast feeding duration		
(i) Never	32	10.7
(ii) <6 months	79	26.3
(iii) 6 months–<1 year	60	20.0
(iv) 1 year–<1.5 year	39	13.0
(v) 1.5–2 year	90	30.0
Bottle feeding duration		
(i) never	62	20.7
(ii) <6 months	76	25.3
(iii) 6 months–<1 year	36	12.0
(iv) 1 year–<1.5 year	33	11.0
(v) 1.5–2 year	93	31.0
Solid food introduction in age		
(i) <6 months	104	34.7
(ii) 6 months or more	196	65.3
Number of meals/day		
(i) 1 meal	12	4.0
(ii) 2 meals	87	29.0
(iii) 3 meals	190	63.3
(iv) 4 meals	11	3.7
Number of snacks/day		
(i) 1 snack	82	27.3
(ii) 2 snacks	154	51.4
(iii) 3 snacks	55	18.3
(iv) 4 snacks	9	3.0

**Table 5:** Distribution of studied children according to their nutritional status.

Anthropometric measurement	Nutritional status	No	%	Minimum	Maximum
Weight/age	Under weight	0	0.00	−1.48	1.58
	Normal	300	100.00		
	Over weight	0	0.00		

Height/age	Stunted	0	0.00	−1.28	1.65
	Normal	300	100.00		
	Tall	0	0.00		

Weight/height	Wasted	0	0.00	1.59	1.23
	Normal	300	100.00		
	Over weight	0	0.00		

Total			300	100,00	

**Table 6:** Factors significantly affecting consumption of healthy food items (vegetables, fruits, and legumes) in preschool study population children.

Vegetables							
	Rare (*n* = 28)		Infrequent (*n* = 29)		Frequent (*n* = 243)		Test of significance (*P* value)
	No	%	No	%	No	%	
Number of meals/day							
(i) 1 meal	5	17.90	3	10.30	4	1.60	*X* ^2^ = 32.10 (*P* = 0.00)
(ii) 2 meals	8	28.90	15	51.70	64	26.30	
(iii) 3 meals	15	53.60	11	37.90	164	67.50	
(iv) 4 meals	0	00.00	0	00.00	11	4.50	

Fruits							
	Rare (*n* = 20)		Infrequent (*n* = 22)		Frequent (*n* = 258)		Test of significance (*P* value)
	No	%	No	%	No	%	

Mother work							
(i) Housewife	16	80.80	12	54.40	130	50.40	*X* ^2^ = 6.56. (*P* = 0.038)
(ii) Working	4	20.00	10	46.60	128	49.60	
Type of the family							
(i) Nuclear	9	45.00	16	72.70	188	72.90	*X* ^2^ = 7.03 (*P* = 0.03)
(ii) Extended	11	55.00	6	27.30	70	27.10	
Number of snack/day							
(i) 1 snack	6	30.00	15	68.20	61	23.60	*X* ^2^ = 26.90 (*P* = 0.00)
(ii) 2 snacks	12	60.00	4	18.20	138	53.50	
(iii) 3 snacks	2	10.00	1	4.50	52	20.20	
(iv) 4 snacks	0	00.00	2	9.10	7	2.70	

Legumes							
	Rare (*n* = 147)		Infrequent (*n* = 50)		Frequent (*n* = 103)		Test of significance (*P* value)
	No	%	No	%	No	%	

City							
(i) Qatif	85	57.80	19	38.00	46	44.70	*X* ^2^ = 7.65 (*P* = 0.00)
(ii) Dammam	62	42.20	31	62.00	57	55.30	
Number of meals							
(i) 1 meal	9	6.10	1	2.00	2	1.90	*X* ^2^ = 13.18 (*P* = 00.04)
(ii) 2 meals	45	30.60	6	12.00	36	35.00	
(iii) 3 meals	88	59.90	40	80.00	62	60.20	
(iv) 4 meals	5	3.40	3	6.00	3	2.90	

**Table 7:** Factors significantly affecting consuming unhealthy food items (pizza, burger, and soft drinks) in preschool study population children.

Pizza							
	Rare (*n* = 148)		Infrequent (*n* = 73)		Frequent (*n* = 79)		Test of significance (*P* value)
	No	%	No	%	No	%	
Type of the family							
(i) Nuclear	92	62.20	61	83.60	60	75.90	*X* ^2^ = 12.14 (*P* = 0.002)
(ii) Extended	56	37.80	12	16.40	19	24.10	

Burger							
	Rare (*n* = 177)		Infrequent (*n* = 47)		Frequent (*n* = 76)		Test of significance (*P* value)
	No	%	No	%	No	%	

City							
(i) Qatif	96	54.20	16	34.00	38	50.00	*X* ^2^ = 6.05 (*P* = 0.048)
(ii) Dammam	81	45.80	31	66.00	38	50.00	
Type of the family							
(i) Nuclear	112	63.30	39	83.00	62	81.60	*X* ^2^ = 12.53 (*P* = 0.002)
(ii) Extended	65	36.70	8	17.00	14	18.40	

Soft drinks							
	Rare (*n* = 202)		Infrequent (*n* = 26)		Frequent (*n* = 72)		Test of significance (*P* value)
	No	%	No	%	No	%	

City							
(i) Qatif	108	53.5	15	57.7	27	37.5	*X* ^2^ = 6.08 (*P* = 0.048)
(ii) Dammam	94	46.5	11	42.3	45	62.5	
Number of meals							
(i) 1 meal	10	5.00	0	0.000	2	2.80	*X* ^2^ = 20.77 (*P* = 0.002)
(ii) 2 meals	43	21.30	9	34.60	35	48.60	
(iii) 3 meals	141	69.80	16	61.50	33	45.80	
(iv) 4 meals	8	4.00	1	3.80	2	2.80	
Number of months in nursery/year							
(i) 0	149	73.80	15	57.70	50	69.40	*X* ^2^ = 33.38 (*P* = 0.015)
(ii) 3–5	2	10.00	17	65.40	0	0	
(iii) 6–8	28	13.90	5	19.20	12	16.70	
(iv) 9–12	23	11.50	4	15.30	10	12.60	

**Table 8:** Factors significantly affecting consuming unhealthy food items (pizza, burger, and soft drinks) in preschool study population children.

Biscuit							
	Rare (*n* = 29)		Infrequent (*n* = 36)		Frequent (*n* = 235)		Test of significance (*P* value)
	No	%	No	%	No	%	
Total family income in Saudi Riyals (S.R)							
(i) less than 2000	1	3.40	0	00.00	9	3.80	*X* ^2^ = 15.75 (*P* = 0.049)
(ii) 2000–4999	7	24.10	0	00.00	47	20.00	
(iii) 5000–9999	11	37.90	14	38.90	79	33.60	
(iv) 10000–14999	2	6.90	12	33.33	45	19.10	
(v) 15000 or more	8	27.60	10	27.80	55	33.40	

Chocolate							
	Rare (*n* = 54)		Infrequent (*n* = 45)		Frequent (*n* = 201)		Test of significance (*P* value)
	No	%	No	%	No	%	

Child attending nursery or kindergarten							
(i) Yes	8	14.80	15	33.30	63	31.30	*X* ^2^ = 6.25 (*P* = 0.04)
(ii) No	46	85.20	30	66.70	138	68.7	
T.V watching hours/day							
(i) less than 2 hours	34	63.00	27	60.00	89	44.30	*X* ^2^ = 11.11 (*P* = 0.025)
(ii) 2 hours or more	20	37.10	18	40.00	112	55.70	

Chips							
	Rare (*n* = 46)		Infrequent (*n* = 39)		Frequent (*n* = 215)		Test of significance (*P* value)
	No	%	No	%	No	%	

Mother work							
(i) Housewife	25	54.30	13	33.30	120	55.80	*X* ^2^ = 6.75 (*P* = 0.034)
(ii) Working	21	45.70	26	66.70	95	44.20	
T.V watching hours/day							
(i) less than 2 hours	32	69.60	21	53.80	97	45.10	*X* ^2^ = 14.39 (*P* = 0.006)
(ii) 2 hours or more	14	30.00	18	46.20	118	54.90	
